# Health Tract, No. 235

**Published:** 1865

**Authors:** 


### HEALTH TRACT, No. 235.

### PROVIDENCE.

Is a "seeing before," is a provision for the future; it is a word commonly employed as an attribute of the Almighty, in reference to that affectionate superintendence which he exercises over the creatures he has made, especially those who consistently endeavor to put their trust in him for guidance, protection, and support as to the affairs of life; and thrice happy are they who have an immovable faith in such a care, whose whole souls go lovingly out in the expression:

"In each event of life how clear
Thy ruling hand I see!"

Those who most observe providences will have most providences to observe. These are the happy ones who learn at length to find light in darkness, joy in sorrow, and good in every thing; these are they whose souls are always tranquil; they have no tumults, no shocks, no boisterous griefs:

"Swift as their thoughts their joys come on,
But fly not half so swift away;
Their souls are ever bright as noon,
And calm as summer evenings be."

Nine tenths of human sorrows arise from having a will different from that of the Almighty. Those who have not learned in faith and love to observe providences, find innumerable sources of irritation in what occurs around them; they can't bear to be thwarted in any thing; they know no will but their own, hence often find themselves "grinding in the prison-house" of impotent insubordination. To them the sun never shines as it ought to; to them the summer shower, which all nature greets with gladness, never falls at the right time; and there grows up in them a "root of bitterness;" no joyousness in the countenance, no glad word upon the lips, no smile upon the face, no mirth in the eye. Such people are never well, either in body or mind; and this is the point of the article, the cultivation of an habitual faith in the good providence of God as a means of physical health in giving ability to stand up under life's trials with a high moral courage, which sees a good in them in the distance, thus enabling them to battle manfully and hopefully against every opposition, against every obstacle, and against every discouragement. The thought of wife, children, and home many a time keeps life in the shipwrecked sailor at sea, and nerves him up to efforts which save him, and which otherwise would never have been made. Thus an habitual and loving faith that there is behind the clouds of life a smiling providence, imparts that moral courage which is the very element of success in life—an element which belongs to every man who is the architect of his own fortunes; these are they who are "the men of their time," who stand out as the best supports of society and helpers of their kind. Thus waiting on Providence and loving their neighbor and living to purpose, they beautifully demonstrate that "godliness is profitable unto all things, having promise of the life that now is, and of that which is to come."

